# A Visit of Inspection

**Published:** 1916-10-07

**Authors:** 


### A Visit of Inspection.

We have received the following account of a visit of inspection on behalf of one of the large "Funds" from a reliable source, together with the assurance that, though perhaps not literally true, it is well founded on fact. Be that as it may, we print the account of the incident as a warning to other institutions, and a reminder to all hospital employees of the importance of precautions against fire and of fire-drill.

Scene: A London Hospital.

*Personæ: Representatives of a Hospital Fund Committee and various Members of the Staff.*

*They make inquiries into the Fire Regulations, etc., and obtain the following varied information from:—*

Wardmaid: "Oh, bless you! I can't be wasting my time on that! It's all written up there on those big cards if you want to see it."

Nervous New Pro.: "I'm sorry, but I really don't know. I haven't got to that yet. I have only been here a week."

Sister: "Oh! the porters see to all that kind of thing. I always 'phone for them."

Night Staff's Quarters.—Hearing the alarm rung, a few tired nurses put their heads out of their doors, but, seeing strangers, retire hastily.

In final despair the Representatives ring the alarm into the R.M. Officer's room. This worthy, in a very annoyed manner, rings up front-hall porter to inquire "Who the dickens is bothering me like this?"

Hall Porter (in a soothing tone): "Never you mind, Sir—it's only them gentlemen a-trying things!"

Collapse and retreat of the Fund Visitors. Their report, when received, was not conducive to a calm atmosphere in the hospital that day!

